# Spontaneous Respiration Using Intravenous Anesthesia and High-Flow Nasal Oxygen (STRIVE Hi) Management of Acute Adult Epiglottitis: A Case Report

**DOI:** 10.1213/XAA.0000000000000635

**Published:** 2017-09-26

**Authors:** Phillip Kwan-Giet Lee, Anton Willis Gerard Booth, Kim Vidhani

**Affiliations:** From the University of Queensland, Princess Alexandra Hospital, Brisbane, Queensland, Australia.

## Abstract

High-flow nasal oxygen (HFNO) is a potentially life-saving adjunct in the emergency management of the obstructed airway. HFNO has multiple beneficial applications in critical care and respiratory support, but its use in emergency-obstructed airway management has not been defined. This case report describes spontaneous respiration using intravenous anesthesia and high-flow nasal oxygen to successfully manage acute adult epiglottitis with rapidly progressing airway obstruction. Oxygenation, carbon dioxide levels, and airway patency were maintained, which facilitated endotracheal intubation while the patient was spontaneously breathing during general anesthesia. The application of HFNO can be extended to emergency airway obstruction.

## Abstract 2

Supplemental Digital Content is available in the text.

Spontaneous respiration using intravenous anesthesia and high-flow nasal oxygen (STRIVE Hi) is a new airway management technique combining the benefits of high-flow nasal oxygen (HFNO) with spontaneous ventilation. It has been shown to preserve airway patency, oxygenation, and carbon dioxide levels in elective cases with severe airway obstruction and respiratory compromise.^1^ This is the first description of its use managing an airway emergency. Acute adult epiglottitis is an emergency where immediate prognosis relates to the ability to provide controlled airway protection. This case demonstrates that STRIVE Hi can be extended to the emergency management of the obstructed airway with a potential increase in margin of safety. Written patient consent was obtained for this publication.

## CASE DESCRIPTION

A 54-year-old, 65-kg man was brought to the emergency department (ED) by ambulance with stridor and difficulty breathing after 2 days of sore throat, odynophagia, and voice change. Significant history included intravenous drug use, hepatitis C virus infection, and homelessness. He was febrile (temperature 38.1°C), with a heart rate of 98 beats/min, blood pressure of 111/76 mm Hg, respiratory rate of 22 breaths/min, and oxygen saturation of 92% on room air. On initial presentation to the ED, the patient’s stridor had resolved and he displayed only mild respiratory distress after treatment with nebulized epinephrine in the ambulance.

A provisional diagnosis of acute epiglottitis was made, and the otolaryngology and anesthesiology departments were consulted. A flexible nasendoscopy was performed, which confirmed the diagnosis, and further nebulized epinephrine with intravenous hydrocortisone and ceftriaxone was administered. Despite these measures, the patient deteriorated while in the ED. He became stridulous again and needed to sit forward with increasing respiratory distress. A joint decision was made by the otolaryngology, anesthesiology, and emergency teams to secure the patient’s airway in the operating room.

Airway assessment demonstrated a Mallampati score II, thyromental distance >6 cm, poor dentition, good mouth opening and neck range of movement, and an easily palpable cricothyroid membrane.

A STRIVE Hi technique was used to induce general anesthesia.^1^ HFNO (Optiflow THRIVE; Fisher and Paykel Healthcare, Auckland, New Zealand) was commenced at 50 L/min and increased to 70 L/min during the induction. A target-controlled infusion of propofol was commenced at a plasma target of 2 μg/mL and titrated upward to a plasma target of 5 μg/mL, using a previously described protocol that maintains spontaneous ventilation.^1^ Local anesthetic was sprayed into the patient’s oropharynx. Indirect laryngoscopy using a C-MAC videolaryngoscope with a size 4 standard Macintosh blade (Karl Storz, Tuttlingen, Germany) was then performed. An initial attempt, performed by a training registrar, revealed a grossly swollen epiglottis and arytenoids without any visible glottic opening. In a second attempt performed by a consultant anesthesiologist, the videolaryngoscope blade was placed beneath the epiglottis (in the direction of bubbling secretions) and then lifted to expose a posterior glottic view (Supplemental Digital Content, Video 1, http://links.lww.com/AACR/A132). The trachea was intubated with a size 6 endotracheal tube railroaded over a bougie. Spontaneous ventilation was maintained throughout the induction and intubation without reactivity or coughing. The minimum oxygen saturation was 96%, and the end-tidal carbon dioxide was 39 mm Hg after intubation. Otolaryngologic surgeons were scrubbed and ready to perform an emergency tracheostomy if the induction or intubation failed or if the intubation was deemed to be impossible during videolaryngoscopy.

The patient was transferred to the intensive care unit for ongoing management. Pharyngeal swabs isolated *Streptococcus milleri*. An elective surgical tracheostomy was performed the following day due to progressive airway edema that had not responded satisfactorily to antimicrobial and steroid therapy. He was decannulated and discharged from hospital 11 days later.

## DISCUSSION

The versatility of HFNO in critical care has accelerated its uptake into the domain of difficult airway management in anesthesia. Having described the use of STRIVE Hi during the elective management of severely obstructed airways,^1^ we now report its use in a well-known airway emergency, which also satisfies one of the prime American Society of Anesthesiologists difficult airway algorithm requirements: preservation of spontaneous breathing.^2^

Acute adult epiglottitis is a true airway emergency with an incidence of 1–4 per 100,000 and an estimated mortality of 0.9% in the United States.^3–5^ Although the definitive treatment of epiglottitis requires administration of broad-spectrum antibiotics, airway protection determines the initial prognosis and remains the immediate priority.^6^ There is, however, no consensus about the optimal method or timing of airway intervention, which creates several dilemmas for those involved in its clinical management.

The variable presentation of adult epiglottitis along with its unpredictable course and potential for rapid progression (as illustrated by this case report) can also add to the management dilemma. The clinical spectrum ranges from sore throat or voice change to stridor with respiratory distress. In severe cases, infection of the epiglottis and other supraglottic structures can lead to rapid and fatal airway obstruction.^4,6^

In less severe cases, the airway may be managed conservatively, while the patient is closely monitored in a critical care environment.^7^ In more severe cases, a range of predictors for airway intervention exists; however, the timing of this intervention is subject to debate.^5,7,8^

The optimal method to secure an obstructing airway caused by acute epiglottitis is unclear.^7^ Tracheal intubation can be difficult or impossible due to the distorted anatomy, airway narrowing, and profuse secretions. All intubation techniques (rapid sequence induction, awake fiberoptic, or inhalational induction) and use of muscle relaxants can precipitate complete airway obstruction.^3,9,10^

Preservation of spontaneous ventilation is a traditional approach to manage the obstructed airway and is one of the basic management choices in the American Society of Anesthesiologists Practice Guidelines for Management of the Difficult Airway.^2,9^ An inhalational induction is the conventional method to preserve spontaneous ventilation; however, its role in obstructed airway management has recently been questioned due to an association with several failures (including epiglottitis).^11,12^

STRIVE Hi is a new airway management technique that combines the benefits of HFNO with spontaneous ventilation. It has been shown to preserve airway patency, oxygenation, and carbon dioxide levels in patients with severe airway obstruction and respiratory compromise requiring elective surgery^1^; however, this is the first description of it being used to manage an acute airway emergency.

HFNO has multiple beneficial applications in critical care and respiratory support.^13^ During the spontaneous respiration induction of anesthesia, HFNO provides continuous positive airway pressure, which can splint open the upper airway, improve airway patency, and reduce airway resistance.^1,13^ This may be particularly advantageous for acute epiglottitis and increase the margin of safety during intubation compared to traditional techniques.

In this case, STRIVE Hi maintained airway patency, oxygen saturation, and end-tidal carbon dioxide levels in a patient with severe airway and respiratory compromise, which facilitated a controlled airway assessment (using videolaryngoscopy) and subsequent intubation. Preservation of spontaneous respiration also generated bubbling of secretions, which may have aided intubation if there was more pronounced supraglottic edema.^10^ Furthermore, if the airway was deemed impossible to intubate during laryngoscopy, the STRIVE Hi technique could be continued to facilitate a controlled tracheostomy, which is a fundamental advantage of preserving spontaneous ventilation during management of the obstructed airway.^9^

The use of neuromuscular blocking agents has been advocated in recent guidelines to manage the difficult airway; however, their role in acute epiglottitis is contentious.^14^ Although neuromuscular blockade provides optimal conditions for intubation (and ventilation^15^), this has traditionally been contraindicated in epiglottitis due to the risk of precipitating complete airway obstruction from a loss of airway tone.^3,16^

It could be argued that an awake tracheostomy is the safest method to secure the airway in severe upper airway obstruction. There are proponents of routine awake tracheostomy for epiglottitis; however, others consider this unnecessarily invasive.^7^ An emergency awake tracheostomy using local anesthesia is a challenging procedure that can be technically difficult and requires a cooperative patient.^11^ It is associated with an increased rate of early and late complications (up to 38% in some series), compared to elective tracheostomy.^17^ Complete airway obstruction, despite the use of HFNO, has recently been described.^18^

As with all techniques, STRIVE Hi has several potential disadvantages. In the absence of neuromuscular blockade, laryngoscopy conditions may be suboptimal and there is a risk of airway reactivity including laryngospasm during intubation or the direct application of local anesthesia. In this case, local anesthesia was sprayed into the oropharynx for dispersion in the upper airway by the HFNO to minimize reactivity. Similarly, conditions for emergent conversion to tracheostomy may also be suboptimal. An otolaryngologic surgeon should be immediately available to perform a tracheostomy in the event of failed intubation or complete airway obstruction.

These potential limitations are balanced by the advantage that spontaneous ventilation has to provide ongoing gas exchange during management of an acutely obstructed airway.^19^ The airway management of epiglottitis may vary at different institutions and will be influenced by contextual factors,^19^ including: (1) the severity of obstruction, (2) patient characteristics (airway assessment, ability to cooperate), (3) equipment availability, and (4) familiarity with a particular technique.

We recommend gaining experience with STRIVE Hi in an elective setting before its use in an obstructed airway emergency. Our standardized protocol to titrate propofol has been reproducible with consistent end points for airway instrumentation without apnea in over 100 cases. We have also described equivalent methods to titrate propofol without target-controlled infusion.^20^ Ketamine may be an alternative to propofol to maintain spontaneous ventilation; however, the lack of titratability and sialogogue effects are potential disadvantages.

This case also highlights that the most-experienced anesthesiologist should manage a severe airway emergency, such as epiglottitis. The difficulty in navigating through distorted airway anatomy resulted in multiple attempts at intubation that had the potential to worsen airway edema and obstruction. While providing an excellent learning opportunity, this needs to be balanced against the potential for rapid clinical deterioration.

STRIVE Hi is a modern alternative to the traditional inhalational induction that has significant potential to improve management of the difficult or obstructed airway.^1^ This case demonstrates that the application of HFNO can be successfully extended to acute airway emergencies, such as adult epiglottitis.

## DISCLOSURES

**Name:** Phillip Kwan-Giet Lee, MBBS, BSc, FANZCA.

**Contribution:** This author helped the supervising anesthesiologist for this case and helped write the manuscript.

**Conflicts of Interest:** None.

**Name:** Anton Willis Gerard Booth, MBBS, FANZCA.

**Contribution:** This author helped write the manuscript.

**Conflicts of Interest:** Anton Willis Gerard Booth is director of a company (Specialist Airway Solutions Pty Ltd), which holds a US patent for an airway intubating device, unrelated to this manuscript and has received travel and accommodation funding from Fisher and Paykel Healthcare. His partner (Renae King) also has a shareholding in Specialist Airway Solutions.

**Name:** Kim Vidhani, MBChB, FRCA, FCICM, FANZCA, FFICM.

**Contribution:** This author helped write the manuscript.

**Conflicts of Interest:** None.

**This manuscript was handled by:** Raymond C. Roy, MD.

## Supplementary Material

**Video 1.** The shoulder shrug maneuver. The video highlights a new technique to facilitate delivery during shoulder dystocia. Video created by Ricardo Leante, MS. Used with permission.
